# Plant health emergencies demand open science: Tackling a cereal killer on the run

**DOI:** 10.1371/journal.pbio.3000302

**Published:** 2019-06-03

**Authors:** Sophien Kamoun, Nicholas J. Talbot, M. Tofazzal Islam

**Affiliations:** 1 The Sainsbury Laboratory, University of East Anglia, Norwich Research Park, Norwich, United Kingdom; 2 Department of Biotechnology, Bangabandhu Sheikh Mujibur Rahman Agricultural University, Gazipur, Bangladesh

## Abstract

Outbreaks of emerging plant diseases and insect pests are increasing at an alarming rate threatening the food security needs of a booming world population. The role of plant pathologists in addressing these threats to plant health is critical. Here, we share our personal experience with the appearance in Bangladesh of a destructive new fungal disease called wheat blast and stress the importance of open-science platforms and crowdsourced community responses in tackling emerging plant diseases. Benefits of the open-science approach include recruitment of multidisciplinary experts, application of cutting-edge methods, and timely replication of data analyses to increase the robustness of the findings. Based on our experiences, we provide some general recommendations and practical guidance for responding to emerging plant diseases.

## Abstract 2

In this Perspective article, the authors share their personal experience with the appearance in Bangladesh of a destructive new fungal disease called wheat blast and stress the importance of open science platforms and crowdsourced community responses in tackling emerging plant diseases.

Wheat likely isn’t the first thing that comes to mind when we think of the densely populated south Asian country of Bangladesh. Yet wheat has become the second largest food crop in the country, after rice, helping Bangladeshi farmers feed their 160 million countrymen. Wheat production has grown from approximately 0.1 million in the 1970s to 1.4 million tons in 2015. Domestic consumption had also risen to about 6 million tons per annum with wheat being an important ingredient of Bengali cuisine. To meet the gap, Bangladesh imported wheat in large amounts—and this is where the problems began.

The wheat crop in Bangladesh had long been viewed as relatively disease free. Many of the usual wheat diseases familiar to farmers elsewhere in the world, such as Septoria leaf blotch and rust diseases, were seldom reported. Many wheat farmers managed to grow a healthy crop with little need to spray fungicides. Then, in February 2016, the crop failed. Dramatically. An untimely rainfall followed by a sudden outbreak of a new fungal disease devastated 15,000 hectares of wheat in 8 districts with yield loss of up to 100% [1]. Some farmers lost their entire crop and had nothing to harvest. Others were forced to burn their sterile fields to prevent further spread of the pathogen. Unusually hot and humid climatic conditions due to untimely rain fall further exacerbated the epidemic [2].

As news of this destructive outbreak spread out, we volunteered to apply the latest genomics technology to identify the precise nature and origin of the culprit. With the help of instant messaging, 2 of us (Tofazzal and Sophien) coordinated the rapid collection of diseased samples and transcriptome sequencing directly from diseased plant tissue. This approach, known as field pathogenomics, enables swift genetic diagnostic of a pathogen population without time-consuming culturing and purification of the fungus [3]. We elected to publicly release the sequence data of our samples through an open-science web platform that we called OpenWheatBlast (http://www.wheatblast.net). Nick and other plant pathologists volunteered unpublished genome sequence data of the wheat blast pathogen to add to our data and the single genome sequence that was publicly available prior to OpenWheatBlast. Two population geneticists, Daniel Croll and Pierre Gladieux, volunteered to independently analyse the data. Both of their conclusions were unequivocal. The Bangladeshi strain of the wheat blast fungus is closely related to the South American population of the wheat blast pathogen *Magnaporthe oryzae* (Syn. *Pyricularia oryzae*). Based on phylogenomic analyses of these independent groups and our laboratory tests, we concluded that the pathogen was introduced to Bangladesh from South America [1]. This scenario is not that surprising. About 6 months before the outbreak, a scandal broke out in Bangladesh following investigative reporting by local science journalists, notably Iftekhar Mahmud, who found that wheat imported from Brazil was “rotten and infected with fungi” [4]. Given that wheat blast is a seed-transmitted disease, it remains plausible that infected wheat shipments from 2015 somehow made it to agricultural fields a few months later.

## Emerging plant diseases call for a rapid, coordinated response

The wheat blast epidemic is emblematic of the rise in emerging plant diseases [5–7]. As the world is becoming more and more interconnected and global trade spreads, plant pathogens are increasingly invading new continents. Climate change and the propensity of these pathogens to jump from one host to another only exacerbate this problem.

When emergencies such as the wheat blast outbreak take place, we expect scientists to immediately release the data and recruit experts. Sometimes this does happen. But we were taken aback by a rather hostile response from some quarters of the community to our rallying cry for international experts to volunteer their time, funds, and resources to help Bangladesh address the emergency. Fortunately, the agriculture minister of Bangladesh, Matia Chowdhury, was extremely supportive. After listening to Tofazzal the day after the launch of OpenWheatBlast, she was full of pride and praise for the team’s unprompted engagement and call to arms. Nonetheless, this progressive attitude towards open science remains somewhat exceptional among decision makers and scientists alike. Traditional plant pathologists work at a pace that is often set by their desire to publish papers and stake priority claims in discovery. Research groups hold on to data, are careful to withhold findings until they can submit a manuscript, and are not necessarily forthcoming in sharing strains and host varieties. What the wheat blast outbreak has shown is that this situation cannot stand. When a crisis is underway, it needs a swift, coordinated response. If researchers studying Ebola or Zika virus acted in such an unforthcoming manner, they would be jailed—and rightly so. The response to outbreaks of human diseases are immediate and exemplified by open data, public information, and a scientifically informed, coordinated response. This is clearly what is required for wheat blast and other emerging crop diseases that threaten food security. It is for this reason that the International Society for Plant Pathology (ISPP) has recently proposed a code of ethics, which our experience suggests is urgently needed [8]. Immediate data sharing and the communication of accurate information to the general public, farmers, and political leaders are all critical. Local engagement and regular dialogue with journalists and communicators is also vital in our experience (see Table 1).

**Table 1:** Recommendations for responding to plant health emergencies^1^.

Recommendation	Required action
Engage with local experts	It is critical to seek the knowledge of local experts—plant pathologists, growers, and agricultural extension officers—and to work with them at all stages of the outbreak. Only through local engagement can a plant health emergency be addressed.
Alert authorities and the scientific community	Actively alert authorities via local experts, engage social media to alert the scientific community, and rapidly establish a single web-based community resource.
Accurately record all relevant information	Fundamental ethical standards for accuracy and integrity should always be followed, but there is an even greater responsibility to be critical, open, and honest in communicating information concerning a disease outbreak—to other scientists, to the public, and to the relevant authorities. This is critical for an effective, coordinated response.
Adopt open-science standards at all stages of outbreak	Release all information immediately. Ensure quality standards have been assured but do not allow delays in release of information. Be completely open with all unpublished, related information, genetic resources, strains, cultivars, and know-how. Publicise release of information immediately and as widely as possible. Ensure authorities and public are aware of developments at all stages too.
Acknowledge all contributions	A key requirement for open science is trust among researchers. Acknowledging all contributions openly and generously is vital.
Engage positively with politicians and international leaders	Implementation of any disease control strategy requires political action and authority. Engagement is therefore vital and needs local expertise and guidance for international scientists. It is critical, however, and can’t simply be left to others. Scientists need to engage.
Adopt a solutions-based approach to scientific investigations	Scientific projects, grant applications, and publications need to be planned and formulated using a solutions-based philosophy. The question ‘How does this action aid in disease control?’ should be paramount.

^1^Our recommendations are consistent with the code of ethics for plant health emergencies proposed by ISPP (https://www.isppweb.org/newsletters/pdf/48_12.pdf).

**Fig 1:**
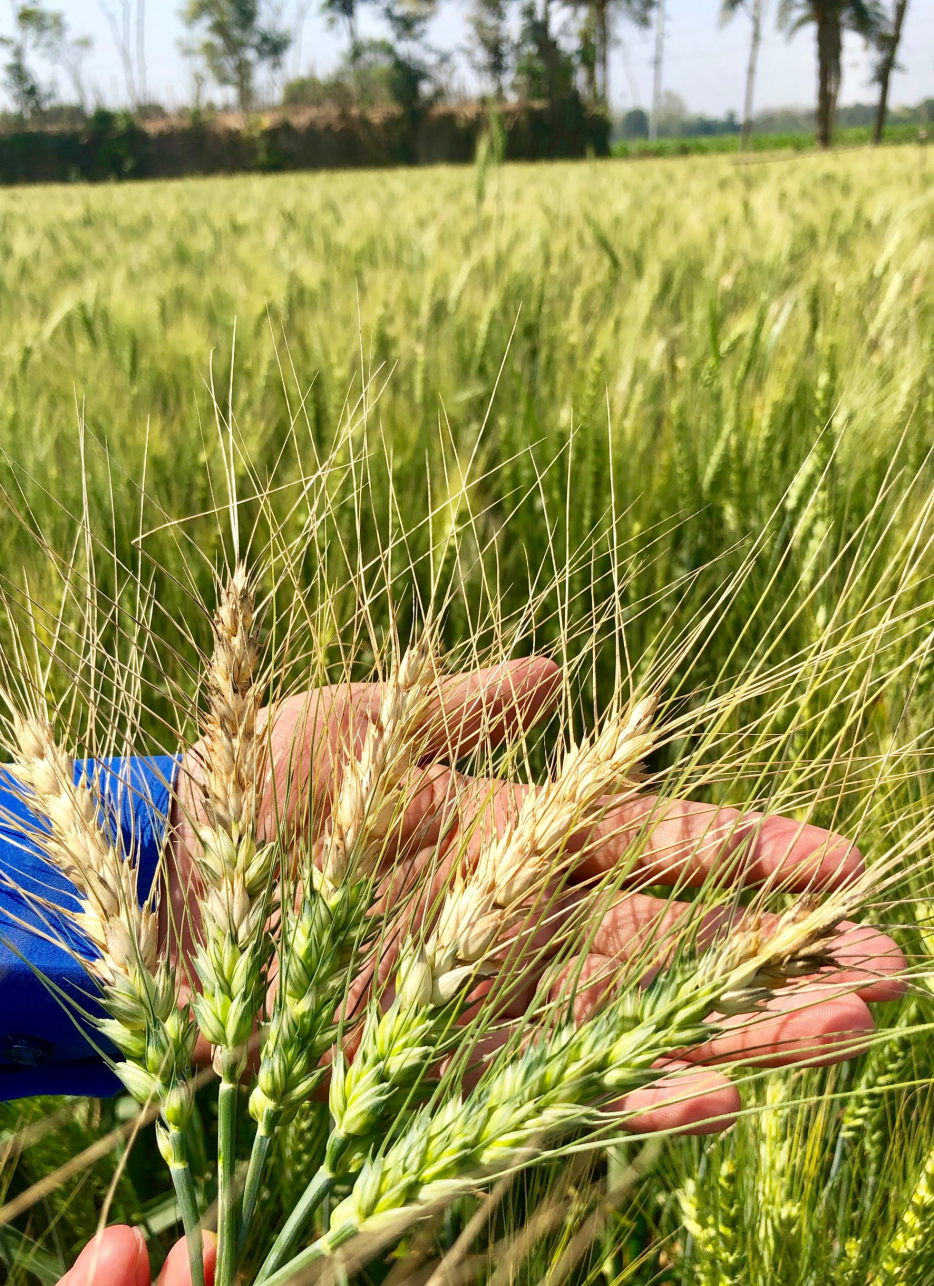
Wheat infected with the blast fungus (February 24, 2019, Meherpur, Bangladesh).

Three years after the 2016 outbreak—just days before we wrote this article—we visited wheat fields in Meherpur near the Bangladesh-India border (Fig 1, S1 Video, S2 Video). This region is now viewed as a hotspot of wheat blast and legitimate concerns that the pathogen would spread over to India and elsewhere remain [2]. Meherpur looks different now than it did prior to the 2016 outbreak. And we have witnessed an unforeseen consequence of blast disease: a food crop has been replaced with a drug crop. Tobacco companies moved in quickly after the wheat blast outbreak, promising farmers guaranteed prices and up-front cash incentives. So now tobacco grows in fields that once grew wheat, and the stench of curing tobacco hangs in the air as children play through racks of drying leaves. A sorry sight.

Although there has not been a repeat of the 2016 epidemic, the disease has spread to 8 more neighbouring districts within a couple of years. The pathogen is clearly now well established in Bangladesh. Wheat blast symptoms are widespread and, in some cases, devastating. It was a powerful and emotional experience for us to stand in the infested fields of Meherpur and hold an empty grainless wheat spike. One of the farmers we visited lost his first sowing in early February 2019 and resorted to harvesting the wheat as fodder long before it set grain. His second sowing at first sight seemed healthy but, despite regular fungicide spraying, signs of wheat blast were evident. As we left Meherpur, a wet weather front was settling in, and we could only brace ourselves for an even more dramatic outbreak.

Wheat cultivation has decreased considerably in the blast-affected districts of Bangladesh to about half of 2016 levels. And in India, West Bengal has declared a 3-year ‘wheat holiday’ following reports of wheat blast in 2017 [2], and the noticeable switch to tobacco cultivation is particularly perverse. But all is not bad. The finding that the pathogen is of the same genetic stock as South American populations meant that the knowledge gathered in Brazil about forecasting, fungicide treatments, and varietal choices can be immediately applied in Asia.

Bangladeshi wheat breeders working with the International Maize and Wheat Improvement Center (CIMMYT) have trialed and released a new wheat variety BARIgom 33 that promises to mitigate the impact of the disease. Other sources of wheat blast resistance, such as the gene *Rmg8*, appear to be effective against a Bangladeshi strain of the fungus [9]. Our own approach is to harness clustered regularly interspaced short palindromic repeats (CRISPR) gene editing technology to breed resistant wheat plants. Already, in collaboration with Emma Wallington at the National Institute of Agricultural Botany, our team has generated the first CRISPR-edited wheat lines, and we have initiated disease resistance tests. In parallel, we continue to monitor the evolution of the pathogen. Given that the Bangladesh population appears to be clonal, we need a high-resolution genome map to precisely define any genetic changes. The near-complete genome sequences of 4 Bangladeshi isolates from 2016 and 2017 that we recently publicly released are a first step in this direction [10]. Throughout these efforts, we have pledged to openly share data, analyses, and bioresources without restriction and prior to formal publication in journal articles.

## Building support for an open-source response

The introduction of wheat blast to Bangladesh is a plant health disaster that has already had both economic and societal consequences for a developing country. We hope that our open-source response has helped to rally the community towards the common goal of fighting this formidable foe and limiting the damage it can cause in Bangladesh and other countries in the region. Our approach has also raised awareness of donors and policy makers for increased funding. The success of our project could, however, also serve as an example for dealing with any future plant health emergency, and we have outlined a set of recommendations and practical actinos, based on our experience in Table 1. Plant pathologists should be ready to embrace open science, given the marked increase in new disease outbreaks [5,7]. However, it is clear that the incentive structures in contemporary academia do not promote open sharing of data ahead of publication. Too much stock is still given to publication of ‘breakthrough’ discoveries of emerging diseases, rather than promoting and, indeed, rewarding actions that could actually lead to more rapid disease control. Only by recognizing these real-world impacts, rather than simple publication metrics, will the situation change. We therefore strongly encourage leaders within universities and institutes to look carefully at these activities and ensure they are captured in promotion criteria and for learned societies and national academies to take a strong stand in reviewing academic incentive structures, so they are fit for purpose. We can be fairly confident that any crop disease that is present anywhere in the world will spread to other areas where climatic conditions allow. Eventually, globalisation will trump phytosanitary inspections, however rigorous they may be. We urgently need to be better prepared and to work proactively to breed locally adapted varieties against diseases that are not yet present in countries around the world. Because the lesson of wheat blast is that these diseases are coming.

We hope that the friendships and collegiality that has developed among our OpenWheatBlast network will inspire other scientists to openly share their data and work together to make the world a better place for us and future generations.

## Supporting information

S1 Video A farmer in Meherpur, Bangladesh, displays grainless wheat spikes in his blast infected field. (MP4)

S2 Video Early harvest of blast infected wheat by a farmer for fodder in Meherpur, Bangladesh. (MP4)

## Abbreviations

CIMMYT: International Maize and Wheat Improvement Center
CRISPR: clustered regularly interspaced short palindromic repeats
ISPP: International Society for Plant Pathology
